# Efficacy and safety of single injection of cross-linked sodium hyaluronate vs. three injections of high molecular weight sodium hyaluronate for osteoarthritis of the knee: a double-blind, randomized, multi-center, non-inferiority study

**DOI:** 10.1186/s12891-017-1591-4

**Published:** 2017-05-26

**Authors:** Chul-Won Ha, Yong-Beom Park, Chong-Hyuk Choi, Hee-Soo Kyung, Ju-Hong Lee, Jae Doo Yoo, Ju-Hyung Yoo, Choong-Hyeok Choi, Chang-Wan Kim, Hee-Chun Kim, Kwang-Jun Oh, Seong-Il Bin, Myung Chul Lee

**Affiliations:** 1 Department of Orthopedic Surgery, Stem Cell and Regenerative Medicine Institute, Samsung Medical Center, Sungkyunkwan University School of Medicine, 81, Irwon-ro, Gangnam-gu, Seoul South Korea; 2 Department of Orthopedic Surgery, Chung-Ang University Hospital, Chung-Ang University College of Medicine, 102, Heukseok-ro, Dongjak-gu, Seoul South Korea; 3 Department of Orthopedic Surgery, Gangnam Severance Hospital, Yonsei University College of Medicine, 211, Eonju-ro, Gangnam-gu, Seoul South Korea; 4 Department of Orthopedic Surgery, School of Medicine, Kyungpook National University, 130, Dongdeok-ro, Jung-gu, Daegu South Korea; 5 Department of Orthopedic Surgery, Chonbuk National University Hospital, Research Institute of Clinical Medicine, Chonbuk National University Medical School, 20, Geonji-ro, Deokjin-gu, Jeonju-si, Jeollabuk-do South Korea; 6 Department of Orthopedic Surgery, Ewha Womans University Mockdong Hospital, 1071, Anyangcheon-ro, Yangcheon-gu, Seoul South Korea; 7 Department of Orthopedic Surgery, National Health Insurance Corporation Ilsan Hospital, 100, Ilsan-ro, Ilsandong-gu, Goyang-si, Gyeonggi-do South Korea; 8 Department of Orthopedic Surgery, Hanyang University College of Medicine, 222-1, Wangsimni-ro, Seongdong-gu, Seoul South Korea; 9 Department of Orthopedic Surgery, Busan Paik Hospital, College of Medicine, Inje University, 75, Bokji-ro, Busanjin-gu, Busan South Korea; 10 Department of Orthopedic Surgery Bundang CHA Hospital, College of Medicine, CHA University, 59, Yatap-ro, Bundang-gu, Seongnam-si, Gyeonggi-do South Korea; 11 Department of Orthopedic Surgery, KonKuk University Medical Center, KonKuk University School of Medicine, 120-1, Neungdong-ro, Gwangjin-gu, Seoul South Korea; 12 Department of Orthopedic Surgery, University of Ulsan, College of Medicine, Asan Medical Center, 88, Olympic-ro 43-gil, Songpa-gu, Seoul South Korea; 13 Department of Orthopaedic Surgery, Seoul National University College of Medicine, 101 Daehak-ro, Jongnogu, Seoul 110-744 Republic of Korea

**Keywords:** Knee osteoarthritis, Inflammation, Treatment, Hyaluronic acid

## Abstract

**Background:**

This randomized, double-blind, multi-center, non-inferiority trial was conducted to assess the efficacy and safety of a cross-linked hyaluronate (XLHA, single injection form) compared with a linear high molecular hyaluronate (HMWHA, thrice injection form) in patients with symptomatic knee osteoarthritis.

**Methods:**

Two hundred eighty seven patients with osteoarthritis (Kellgren-Lawrence grade I to III) were randomized to each group. Three weekly injections were given in both groups but two times of saline injections preceded XLHA injection to maintain double-blindness. Primary endpoint was the change of weight-bearing pain (WBP) at 12 weeks after the last injection. Secondary endpoints included Western Ontario and McMaster Universities Osteoarthritis index; patient’s and investigator’s global assessment; pain at rest, at night, or in motion; OMERACT-OARSI responder rate; proportion of patients achieving at least 20 mm or 40% decrease in WBP; and rate of rescue medicine use and its total consumption.

**Results:**

Mean changes of WBP at 12 weeks after the last injection were −33.3 mm with XLHA and −29.2 mm with HMWHA, proving non-inferiority of XLHA to HMWHA as the lower bound of 95% CI (−1.9 mm, 10.1 mm) was well above the predefined margin (−10 mm). There were no significant between-group differences in all secondary endpoints. Injection site pain was the most common adverse event and no remarkable safety issue was identified.

**Conclusions:**

This study demonstrated that a single injection of XLHA was non-inferior to three weekly injections of HMWHA in terms of WBP reduction, and supports XLHA as an effective and safe treatment for knee osteoarthritis.

**Trial registration:**

ClinicalTrials.gov (NCT01510535). This trial was registered on January 6, 2012.

## Background

In the treatment of mild to moderate osteoarthritis (OA), pharmacologic treatments including analgesics and non-steroidal anti-inflammatory drugs (NSAIDs) are recommended along with various non-pharmacologic modalities such as exercise and weight reduction. However, most of the currently available pharmacologic treatment has its own limitations regarding tolerability and durability. In the search of an alternative treatment option, attention has turned to viscosupplementation, of which the concept was based on the recognition that synovial hyaluronan (HA) is decreased in its concentration and length of its chain in OA patients [1]. HA is a natural viscoelastic substance known to play an important role in lubricating, shock-absorbing, and maintaining the normal physiology of articular cartilage [2].

Since late 1990s, many studies have investigated the efficacy and safety of various intra-articular HA (IAHA) preparations of various molecular weights (MW), especially for knee OA. The compiled available evidence [3–5] suggests positive effect of IAHA preparations in pain reduction and functional improvement and a conditional recommendation was given for IAHA to treat knee OA by professional guidelines [6, 7]. Still, some controversies exist on the clinical efficacy of the intra-articular injection of various HA preparations [8–12]. Therefore, further investigations to gather qualitative data from randomized, controlled trials of various comparisons are warranted.

In the light of this context, we had planned a randomized, double-blind comparative study to investigate the efficacy and safety of a cross-linked HA (XLHA) compared with those of a linear high molecular weight HA (HMWHA). Increase in the MW, stability, and viscosity of HA by cross-linking has been known to result in an extended duration of action with less number of intra-articular injections [13–15]. The present study was therefore designed to assess the non-inferiority of the single injection of XLHA to three weekly injections of HMWHA and to compare the efficacy and safety of the two preparations in patients with mild to symptomatic moderate knee OA.

## Methods

### Materials

A newly developed XLHA product with MW ≥ 10000 kDa, LBSA0103 (LG life sciences, Iksan, South Korea), and Hyruan Plus® (LG life sciences, Iksan, South Korea), a linear HMWHA of mean MW of 3000 kDa were used for this clinical trial. Both preparations are derived from bacteria (*Streptococcus zooepidermicus*). Hyruan Plus® has been used in more than 25 countries with the indications of OA of the knee, shoulder, hip, and ankle although specific indication may be different in each country; and its efficacy and safety have been published in several preclinical [16, 17] and clinical trials [18, 19]. For LBSA0103, HA is modified to form a bigger and more stable HA by using 1, 4-butanediol diglycidyl ether (BDDE) as the cross-linker of which the safety and metabolism have been well established [20].

### Study design

This was a randomized, double-blind, multi-center (12 investigational sites) study to evaluate the efficacy and safety of XLHA compared with HMWHA, in patients with symptomatic knee OA. After going through screening at Visit 1 and washout-period of 2 weeks, eligible patients were randomized into each group (XLHA or HMWHA) in a 1:1 ratio to receive three intra-articular injections of the assigned intervention (Visit 2-Visit 4). Study group received two placebo (saline) and one XLHA injections and control group received three HMWHA injections. The two injection of placebo in the study group was inevitable to maintain the double blindness of this study. A block randomization scheme with a block size of 4, stratified by investigational site and involvement of unilateral or bilateral knees, was generated using SAS® version 9.1 (SAS Institute Inc., Cary, NC, USA). Patients were followed up at 1 week, 6 weeks, and 12 weeks after the last injection (Visit 5-Visit 7). Total study period (FPI-LPO) was 11 months (Dec. 2011-Oct. 2012).

Patients, investigators, and all study related personnel including study monitors were blinded to the assigned treatment. To maintain double-blind conditions, injections were given only by a dedicated independent non-blinded investigator and efficacy assessments were made by another independent investigator in each site, who was also concealed from the treatment information.

The study was coducted in accordance with the ethical principles of Helsinki Declaration, good clinical practice, and applicable local regulations. The government regulatory authority and the IRB of each investigational site granted approval for the study and informed consent was obtained from all individual participants included in the study. The patients who enrolled in this clinical trial did not receive any inappropriate financial compensation except transportation fee.

### Study population

Patients aged 40 years or older with knee OA that satisfies the diagnostic criteria of American College of Rheumatology [21] and Kellgren-Lawrence [22] grade I to III by X-ray were enrolled. All patients underwent simple radiographs including standing anteroposterior and lateral views, standing posteroanterior view with 45° knee flexion (Rosenberg view), and merchant view. The severity of OA based on Kellgren-Lawrence grade was evaluated by two independent experienced radiologists who were blinded to the assigned treatment. The inclusion criteria were Kellgren-Lawrence grade I to III by X-ray, weight-bearing pain (WBP) ≥40 mm in the affected knee by 100 mm visual analog scale (VAS), persistent pain at least in one knee despite of treatments with NSAIDs or other analgesics, and willing to discontinue current OA treatments. The exclusion criteria were the followings: body mass index (BMI) >32; rheumatoid arthritis or other metabolic/inflammatory arthritis; septic arthritis; skin lesion in the affected knee; secondary knee OA; diseases accompanying severe pain such as Sudek’s atrophy, Paget’s disease, or spinal disc herniation; polyarticular OA that may hinder pain assessment of knee; significant loss of patellofemoral joint space confirmed by X-ray; patients with severe knee joint effusion; heart, liver, and kidney disorder; need of anticoagulant medication except aspirin; pregnant or lactating women; women with child-bearing potential not willing to use effective contraception; history of surgical procedures in the affected knee within 12 months; joint replacement surgery of the affected knee; use of previous OA treatments that would interfere with study assessment such as IAHA in the affected knee within 9 months, IAHA in the other joints within 6 months, IA steroid injection within 3 months, or oral steroids within a month; use of contraindicated treatment (refer to 4.4) during washout period; and use of any analgesics including acetaminophen or aspirin within 24 h from randomization; administration of anesthetics within 48 h from randomization.

### Intervention

One dedicated independent investigator per institute performed the injections. The study group received two times of weekly injections of placebo (phosphate buffered saline, 18 mg/2 mL/injection) followed by an injection of XLHA (60 mg/3 mL/injection), on the third week. The control group received three weekly injections of HMWHA (20 mg/2 mL/injection). Injections were given using 21G needles with strict aseptic techniques. Any effusions, if present, were aspirated into a separate syringe before the administration of the XLHA or HMWHA, the same needle was left in place for the XLHA or HMWHA prefilled syringe injection. During the entire study period, patients were not allowed to use steroid, NSAIDs, chondroitin sulfate/glucosamine or other pain relieving methods including physical therapy. Low dose aspirin (≤300 mg/day) taken for a cardiovascular condition was allowed. Acetaminophen (≤4 g/day) taken as a rescue therapy was allowed. However, these agents should be stopped 24 h before each assessment visit.

### Outcome measures

The primary endpoint was the change of weight-bearing pain (WBP) assessed by 100 mm visual analogue scale from baseline (Visit 2) to Visit 7. This variable was also evaluated for Visit 5 and Visit 6 as secondary endpoints. Changes in the following secondary endpoints were also assessed at Visit 5, 6, and 7: Western Ontario and McMaster Universities Osteoarthritis Index (WOMAC) total score; WOMAC subscale (pain, function, stiffness) score; patient’s global assessment; investigator’s global assessment; pain at rest; pain at night; pain in motion; severity of swelling and tenderness; range of knee joint motion at baseline and follow-up visits; the proportion of patients satisfying responder criteria suggested by the Outcome Measures for Rheumatology Committee and Osteoarthritis Research Society International Standing Committee for Clinical Trials Response Criteria Initiative (OMERACT-OARSI) [23]; WBP responder rate, which was defined as the proportion of patients achieving at least 20 mm of decrease or 40% reduction from baseline in WBP; and the proportion of patients using rescue medicine (acetaminophen); total consumption of the rescue medicine.

Safety was assessed by adverse events (AEs). All of the AEs that occured during the study period were collected at each visit by the investigator’s observation and also by asking any experience of AEs to the patients. The AEs were collected regardless of the severity or relationship to the study drugs. The AEs were classified according to the Medical Dictionary for Regulatory Activities (MedDRA Ver. 15.0). Particular attention was paid to injection site reactions (erythema, swelling, pain, or warmth), which was collected by solicitation method using patients’ diary cards for 7 days after each injection. The vital signs and laboratory tests were evaluated and clinically significant change was collected as AEs during the study.

### Statistical analysis

The primary purpose was to prove the non-inferiority of XLHA to HMWHA in terms of decreasing the WBP. Assuming a standard deviation of 24.3 based on the phase III study of HMWHA and the non-inferiority margin of −10 mm [24], the sample size needed for the non-inferiority test was 93 per group, which would provide 80% power at a significance level of 5%. The final sample size was 266 (133 per group) to accommodate 30% drop-out or protocol violation.

For the primary endpoint, two-sided 95% confidence interval (CI) for between-group difference (HMWHA - XLHA) was calculated; and if the lower bound of the CI was greater than -10 mm, the non-inferiority of XLHA to HMWHA was to be declared. Missing values of the primary endpoint were replaced by the last available data according to the last observation carried forward approach. For secondary endpoints, between-group differences and the differences from baseline were tested. Depending on the data normality of continuous variables, a two sample *t*-test or a Wilcoxon's rank sum test was used for the between-group comparison whereas a paired *t*-test or a Wilcoxon’s signed rank test was used for the within-group comparison. For categorical variables, the between-group comparison was tested using a Chi-square test or a Fisher’s exact test. All statistical analyses were performed using SAS® software version 9.1 (SAS Institute, Cary, NC, USA).

## Results

### Patient population

**Fig 1:**
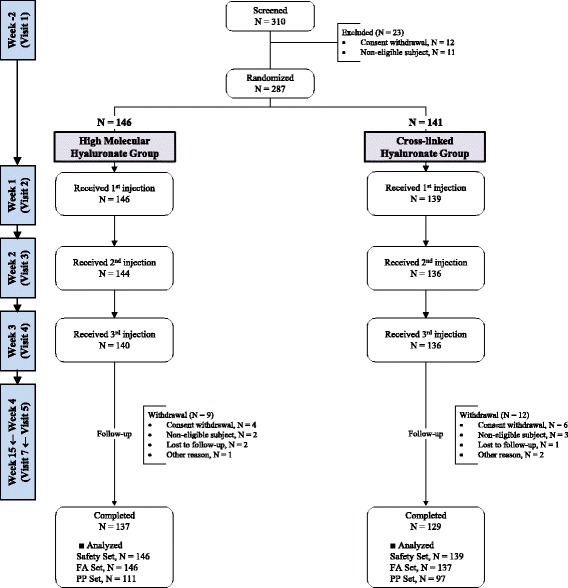
Patient Distribution. The bars in the far *lef*t indicate each visit; however, the distance between each visit is not proportional to the actual time because of the limited space. Patients randomized to the cross-linked hyaluronate (XLHA) group received two placebo injections before the third injections, which was given as XLHA

During the study period, 310 patients were screened and 23 were excluded due to consent withdrawal (12) or non-eligibility (11); 287 were randomized; 276 received planned series of study treatments; 266 completed the 12 weeks of follow-up visits (Fig. 1). Excluding two patients who withdrew their consent before receiving an injection, 285 patients were included in the safety analysis. Among these 285 patients, two were never evaluated for efficacy outcome after baseline and were excluded from the efficacy analysis. Thus full analysis set (FAS) included 283 patients whereas per-protocol set (PPS) included 208 patients after exlcuding 75 patients with major protocol violations. No between-group difference in the drop-out or protocol violation rates was noted (Fig. 1). Patients demographics and baseline characteristics were comparable between the two groups (Table 1).

**Table 1:** Patients demographics and baseline characteristics (Full Analysis Set)

	HMWHA	XLHA	*P*-value
	(*N* = 146)	(*N* = 137)	
Sex			
Male	26 (19%)	32 (22%)	0.54^A^
Female	111 (81%)	114 (78%)	
Age, years	62.38 (8.43)	61.97 (8.64)	0.68^B^
BMI, kg/m^2^	24.82 (2.65)	25.14 (2.91)	0.52^C^
Affected knee, patients			
Left	23 (15.75)	22 (16.06)	0.67^A^
Right	26 (17.81)	30 (21.90)	
Bilateral	97 (66.44)	85 (62.04)	
Duration of knee OA, years			
Left	3.08 (0.08,26)	3.83 (0.08, 22.08)	0.98^C^
Right	3 (0.08, 26)	4 (0.08, 24.08)	0.19^C^
K-L grade, patients			
Grade I	28 (19.18)	19 (13.87)	0.36^A^
Grade II	65 (44.52)	59 (43.07)	
Grade III	53 (36.30)	59 (43.07)	
100 mm VAS on WBP	61.01 (13.22)	61.79 (13.76)	0.68^C^
WOMAC index			
Pain subscore	10.56 (3.31)	10.26 (3.05)	0.33^C^
Function subscore	34.53 (11.64)	33.75 (10.53)	0.56^B^
Stiffness subscore	3.97 (1.72)	3.87 (1.61)	0.72^C^
Total score	49.05 (16.01)	47.88 (14.18)	0.52^C^

*BMI* Body mass index, *OA* osteoarthritis, *K-L* Kellgren-Lawrence, *VAS* visual analogue scale, *WBP* weight bearing pain, *WOMAC* Western Ontario and McMaster Universities Osteoarthritis. Continuous variables are presented with mean (SD) except duration of knee OA, which is presented with median (min, max)

^A^, *P*-value obtained from Chi-square test; ^B^, *P*-value obtained from two-sample *t*-test; ^C^, *P*-value obtained from Wilcoxon’s rank sum test

### Efficacy

**Fig 2:**
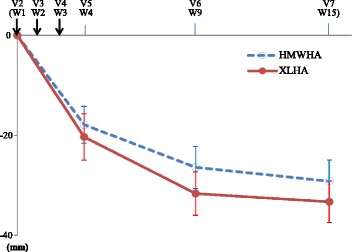
Mean Changes from Baseline in Weight-bearing Pain. Significant reduction in WBP was observed at 1 week after the last injection (−18 mm in HMWHA, *P* < 0.001 and −20 mm in XLHA, *P* < 0.001); and it was further evident at week 6 week and week 12. The changes observed at 12 weeks after the last injection were still significant in both groups (−29 mm in HMWHA, *P* < 0.001 and −33 mm in XLHA, *P* < 0.001). Reductions in WBP at V5, V6 and V7 from baseline were all significant (*P* < 0.001, paired *t*-test) in both groups. V, Visit; W, Week; Vertical arrows represent injections of investigational products; Error bars indicate 95% confidence interval of mean change

The changes in WBP from baseline to Visit 5, 6, and 7 in PPS are shown in Fig. 2. Significant reduction in WBP was observed at 1 week after the last injection and it was further evident at week 6 and week 12. The changes observed at 12 weeks after the last injection were still significant in both groups and the two-sided 95% CI for between-group difference was from −1.9 mm to 10.1 mm. Analysis with FAS also showed similar results: −28 mm in HMWHA and. -32 mm in XLHA; 95% CI, (−1.3 mm, 9.2 mm). As the lower bound of 95% CI was greater than the preset margin (−10 mm), the non-inferiority of XLHA to HMWHA was demonstrated.

Changes in secondary endpoints of continuous variables at the three follow-up visits (Visit 5, 6, and 7) were all significant (*P* < 0.001) and consistently indicated a general improvement with both interventions (Table 2). The magnitude of changes in these secondary endpoints was evident at 6 weeks after the last injection and it was maintained at 12 weeks. No between-group difference was observed in any of the secondary endpoints at every follow-up point. The responder rate by OMERACT-OARSI and by the predefined responder criteria on WBP were slightly higher with XLHA than with HMWHA, although the difference was not statistically significant (Table 3). The severity of swelling or tenderness in the knee joint and the proportion of patients with swelling or tenderness showed meaningful improvement in both groups throughout the follow-up period. No between-group difference was observed (Table 2). The range of knee motion was maintained thoughout the follow-up period and no between-group difference was observed (Table 2). Similar proportions of patients used the rescue medicine: 81/111 (73%) in HMWHA group vs. 62/97 (64%) in XLHA group (*P* = 0.160). The consumption of rescue medication during the study period was similar, and there was a tendency that less proportion of patients in XLHA group used the rescue medicine than those in HMWHA group during the period of Visit 6 – Visit 7 (37% vs. 50%, *P* = 0.053).

**Table 2:** Mean changes in secondary endpoints from baseline to follow-up visits (Per-protocol Set)

		HMWHA	XLHA	*P*-value
		N = 111	N = 97	
WOMAC				
Pain subscore	Visit 5	−3.2 (3.15)	−2.77 (3.28)	0.51^B^
	Visit 6	−3.87 (3.53)	−3.39 (3.64)	0.54^B^
	Visit 7	−4.23 (3.7)	−3.96 (3.37)	0.59^A^
Function subscore	Visit 5	−8.05 (9.73)	−7.89 (10.23)	0.91^A^
	Visit 6	−11.03 (11.84)	−10.74 (10.84)	0.86^A^
	Visit 7	−12.09 (12.47)	−12.57 (9.58)	0.76^A^
Stiffness subscore	Visit 5	−0.99 (1.67)	−0.88 (1.6)	0.58^B^
	Visit 6	−1.21 (1.72)	−1.18 (1.59)	0.94^B^
	Visit 7	−1.34 (1.84)	−1.25 (1.59)	0.75^B^
Total score	Visit 5	−12.18 (13.56)	−11.54 (13.93)	0.74^A^
	Visit 6	−16.02 (16.04)	−15.31 (15)	0.74^A^
	Visit 7	−17.57 (17.21)	−17.77 (13.47)	0.92^A^
Patient’s global assessment				
	Visit 5	−15.11 (18.22)	−17.51 (23.72)	0.91^B^
	Visit 6	−23.15 (21.88)	−24.64 (22.55)	0.86^B^
	Visit 7	−26.91 (24.34)	−30.1 (21.75)	0.32^A^
Investigator’s global assessment				
	Visit 5	−21.86 (20.07)	−21.1 (22.08)	0.71^B^
	Visit 6	−27.66 (19.75)	−28.24 (23.44)	0.93^B^
	Visit 7	−33.23 (20.92)	−32.52 (22.89)	0.82^A^
100 mm VAS on pain				
Rest pain	Visit 5	−12.88 (22.64)	−15.18 (24.51)	0.51^B^
	Visit 6	−20.81 (22.75)	−23.1 (24.43)	0.49^A^
	Visit 7	−21.69 (24.53)	−27.49 (23.69)	0.09^A^
Night pain	Visit 5	−15.07 (23.5)	−13.96 (23.28)	0.87^B^
	Visit 6	−21.83 (26.37)	−23.93 (22.41)	0.54^A^
	Visit 7	−23.28 (27.72)	−25.27 (21.16)	0.56^A^
Motion pain	Visit 5	−15.83 (20)	−17.06 (24.32)	0.59^B^
	Visit 6	−22.67 (23.95)	−25.86 (23.46)	0.34^A^
	Visit 7	−25.93 (26.59)	−29.62 (22.21)	0.28^A^
Range of Knee Motion (°)				
	Visit 5	135.39 (9.07)	134.02 (9.65)	0.27^B^
	Visit 6	135.29 (8.88)	134.35 (9.25)	0.46^B^
	Visit 7	135.71 (8.62)	134.99 (9.23)	0.51^B^
Swelling				
	Visit 5	18 (16.22)	22 (22.68)	0.24^C^
	Visit 6	14 (12.61)	17 (17.53)	0.32^C^
	Visit 7	16 (14.41)	12 (12.37)	0.67^C^
Tenderness				
	Visit 5	33 (29.73)	38 (39.18)	0.15^C^
	Visit 6	31 (27.93)	28 (28.87)	0.88^C^
	Visit 7	24 (21.62)	24 (24.74)	0.59^C^

Data are mean (SD) except for swelling and tenderness, both of which are presented as number of patients (%)

^A^, *P*-value obtained from two-sample *t*-test; ^B^, *P*-value obtained from Wilcoxon’s rank sum test; ^C^, *P*-value obtained from Chi-square test

**Table 3:** Responder rate (Per-protocol Set)

	HMWHA	XLHA	*P*-value^A^
	*N* = 111 (%)	*N* = 97 (%)	
Decrease in weight-bearing pain ≥ 20 mm or ≥ 40% improvement			
Visit 5	52 (46.85)	46 (47.42)	0.93
Visit 6	66 (59.46)	70 (72.16)	0.05
Visit 7	76 (68.47)	73 (75.26)	0.28
OMERACT-OARSI response			
Visit 5	38 (34.23)	28 (28.87)	0.41
Visit 6	48 (43.24)	47 (48.45)	0.45
Visit 7	55 (49.55)	57 (58.76)	0.18

^A^, *P*-value obtained from Chi-square test

### Safety

Patients in both groups well tolerated the interventions. General adverse events were spontaneously reported in 90 patients (32%) during the study: 42 patients (29%) in HMWHA group experienced 63 adverse events whereas 48 patients (35%) in XLHA group experienced 73 adverse events (*P* = 0.295). All general adverse events are summarized by system organ class (Table 4) and adverse events reported in more than 1% of any group at preferred term level were also presented (Table 5). Total of 8 patients experienced 9 adverse drug reactions (ADRs), related with the study interventions; 1 patient (1 event) in HMWHA group, 7 patients (8 events) in XLHA group (Table 6). Between-group difference in the rate of patients experiencing ADR was statistically significant; however, the significance was not relevant after removing 3 events that occurred after placebo injections in XLHA group (0.68% vs. 3.6%, *P* = 0.113, Fisher’s exact test). All those 5 events assessed to be related with XLHA injection did not require medical intervention to treat them; were mild in intensity except one event (joint swelling of moderate intensity); and were resolved during study period except one event (swelling of mild intensity).

**Table 4:** Number of patients experiencing adverse events at system organ class

System Organ Class	HMWHA	XLHA
	*N* = 146 (%)	*N* = 139 (%)
Eye disorders	1 (0.7)	3 (2.2)
Gastrointestinal disorders	9 (6.2)	4 (2.9)
General disorders and administration site conditions	4 (2.7)	9 (6.5)
Infections and infestations	9 (6.2)	13 (9.4)
Injury, poisoning and procedural complications	2 (1.4)	3 (2.2)
Investigations	0 (0)	2 (1.4)
Metabolism and nutrition disorders	1 (0.7)	1 (0.7)
Musculoskeletal and connective tissue disorders	15 (10.3)	12 (8.6)
Neoplasms benign, malignant and unspecified (including cysts and polyps)	0 (0)	1 (0.7)
Nervous system disorders	6 (4.1)	5 (3.6)
Psychiatric disorders	1 (0.7)	0 (0)
Respiratory, thoracic and mediastinal disorders	4 (2.7)	2 (1.4)
Skin and subcutaneous tissue disorders	1 (0.7)	6 (4.3)
Surgical and medical procedures	1 (0.7)	1 (0.7)
Vascular disorders	1 (0.7)	1 (0.7)

**Table 5:** Number of patients experiencing common adverse Events (>1% of Population)

Preferred Term	HMWHA	XLHA
	*N* = 146 (%)	*N* = 139 (%)
Diarrhoea	3 (2.1)	1 (0.7)
Dyspepsia	1 (0.7)	2 (1.4)
Injection site pruritus	2 (1.4)	1 (0.7)
Pain	1 (0.7)	4 (2.9)
Pyrexia	0 (0)	3 (2.2)
Cystitis	2 (1.4)	2 (1.4)
Nasopharyngitis	3 (2.1)	7 (5)
Upper respiratory tract infection	0 (0)	3 (2.2)
Joint swelling	0 (0)	2 (1.4)
Musculoskeletal pain	0 (0)	2 (1.4)
Pain in extremity	6 (4.1)	3 (2.2)
Plantar fasciitis	0 (0)	2 (1.4)
Headache	5 (3.4)	1 (0.7)
Paraesthesia	0 (0)	2 (1.4)
Cough	3 (2.1)	0 (0)
Erythema	0 (0)	2 (1.4)

**Table 6:** Adverse drug reactions

Adverse Drug Reactions	HMWHA	XLHA	*P*-value
	*N* = 146	*N* = 139	
Injection site pruritus	1 (1)	1 (1)^A^	1.00
Pain	0 (0)	1 (2)^A^	0.49
Swelling	0 (0)	1 (1)	0.49
Injection site hemorrhage	0 (0)	1 (1)	0.49
Joint swelling	0 (0)	1 (1)	0.49
Erythema	0 (0)	2 (2)^A^	0.24
Total number	1 (1)	7 (8)	0.03

Data are number of patients experiencing adverse drug reaction and the numbers in parenthesis are number of events

^A^, In XLHA group, 3 adverse drug reactions (each event of injection site pruritus, pain, and erythema) occurred after placebo injection, which preceded XLHA injection

The incidence of local injection site reactions which occurred within for 7 days after each injection were similar in the two groups (*P* = 0.29-0.94, (Table 7)). The proportions of patients experiencing local reactions after HMWHA administration did not differ from those after placebo administration in the XLHA group. In addition, the rates did not increase with the subsequent injection of XLHA on the third week. Those local reactions were mild or moderate in most of the patients (80%-100%) in both groups and lasted three to four days. No patients required a medical treatment to treat them and none were dropped out because of the local reactions. Two serious adverse events (1 rib fracture, 1 malignant neoplasm of lung) were reported during the study period and none were assessed as related with study interventions. No clinically significant changes were found in vital signs, hematology, serum chemistry, and urinalysis.

**Table 7:** Number of patients experiencing solicited local reactions at the injection site

	HMWHA	XLHA	*P*-value
	*N* = 146 (%)	*N* = 139 (%)	
After 1^st^ Injection	58 (39.7)	52 (37.4)	0.69
Redness	27 (18.5)	21 (15.1)	0.45
Swelling	13 (8.9)	10 (7.2)	0.60
Pain	38 (26)	44 (31.7)	0.29
Warmth	23 (15.8)	23 (16.5)	0.86
After 2^nd^ Injection	43 (29.9)	40 (29.4)	0.93
Redness	18 (12.5)	16 (11.8)	0.85
Swelling	6 (4.2)	5 (3.7)	0.83
Pain	30 (20.8)	27 (19.9)	0.84
Warmth	16 (11.1)	13 (9.6)	0.67
After 3^rd^ Injection	38 (27.1)	40 (29.4)	0.68
Redness	17 (12.1)	14 (10.3)	0.63
Swelling	11 (7.9)	11 (8.1)	0.94
Pain	31 (22.1)	34 (25)	0.58
Warmth	12 (8.6)	17 (12.5)	0.29

## Discussion

In this randomized, double-blind, controlled trial in 285 patients with symptomatic knee OA, a single intra-articular injection of XLHA was not inferior to three weekly injection of HMWHA on efficacy with favorable safety profile. Patients in both groups showed significant improvement from baseline throughout the 12-week follow-up period with regard to WBP, pain in various activities, knee function, WOMAC total score and subscale scores, patient’s and investigator’s global assessments, and severity of swelling and tenderness in the knee joint. No significant between-group difference in the secondary efficacy endpoints was observed. Although statistical significance was not identified, it was noteworthy that OMERACT-OARSI responder rate (59% vs. 50%, *P* = 0.184) and WBP responder rate (75% vs. 68%, *P* = 0.279) were slightly higher with XLHA than with HMWHA.

The results of this study showed significant clinical improvement with both treatments at 6 weeks after the last injection. The improvement at 6 weeks was greater than that observed at 1 week after the last injection, and maintained until 12 weeks after the last injection. This course of changes in the clinical efficacy is in agreement with a meta-analysis of 54 clinical trials that included data from 7545 patients [4], which showed IAHA was efficacious by 4 weeks, reached its maximum efficacy by 8 weeks, and its efficacy was maintained up to 24 weeks. Likewise, the degree of change in WBP observed in the present study (−33.3 mm with XLHA and −29.2 mm with Hyruan Plus® at 12 weeks) was similar to the previous studies using HMWHA [19] or other high molecular weight (1000–7000 KDa) HA preparations (Orthovisc® [25] and Artzal® and Synvisc® [26]). In the study with Orthovisc®, IAHA was compared with methyl prednisolone acetate (MPA) and the results in terms of all pain scores and Lequesne index at 3 month favoured IAHA but no between-group difference was found at 6 month. A BDDE cross-linked HA preparation (Durolane®) similar to XLHA was investigaed in patients with knee OA against MPA; its efficacy in terms of WOMAC pain responder rate at 12 weeks was non-inferior to that of MPA; and the benefit of IAHA was maintained up to 26 weeks while that of MPA was reduced [27]. It is noteworthy that a single injection of the cross-linked HA (60 mg) was enough to maintain its effect until 26 weeks whereas repeated injections of the linear HA (30 mg/injection) were needed to obtain similar efficacy brought by the same number of injections of MPA [27]. Unlike the un-modified HA, which undergoes rapid degradation enzymatically [28] or by oxidation [29], the cross-linked HA has slow degradation rate and increased elastoviscous properties, thus extended durability at the injection site [1]. As injection procedure itself bears risks of infection, pain and/or bleeding, reducing the injection numbers while obtaining comparable efficacy is a reasonable ground for choice of a cross-linked HA unless safety issues come up with cross-linking agents. The LBSA0103 is produced using BDDE as the cross-linking agent, which has reactive epoxide groups in either side of chain but these are neutralized after forming stable ether bonds with alcohol in the HA backbone [30] and the amount of unreacted BDDE is negligible. Therefore, safety risk associated with chemical states of BDDE present after cross-linking reaction is very low and the safety and metabolism of BDDE cross-linked HA have been well established.

No remarkable safety issue was identified in this study and the safety profile obtained was consistent with those observed with other IAHA preparations [31]. The incidence of solicited local injection site reactions and the incidence of treatment-emergent adverse events were similar in both groups. Both IAHA preparations used in this study are non-animal origin, which may have contributed to the favourable safety profile due to low risk of developing immunological response. In a double-blind, randomized study comparing fermented (bacterial origin) HA with avian origin HA, injection site effusions were less frequent with fermented HA than with avian-origin HA (0.6% vs. 8.1%, *P* = 0.002) [32].

Despite the wealth of available data, efficacy of IAHA is still in debate and available guidelines [6, 7, 33] do not present consistent recommendation. Several studies using placebo comparator showed contradictory results and this made the data interpretation challenging [8, 9, 29, 34–38]. In a study by Jørgensen et al. [35], 337 patients with moderate to severe knee OA received five injections of a low MW HA (Hyalgan®) or saline and were followed up at 3, 6, 9 and 12 months after the treatment. But IAHA did not show better improvement in pain, function, acetaminophen consumption, or other efficacy parameters than saline injections. Another study where the same product was compared with placebo and naproxen (500 mg orally twice a day) in 495 patients with knee OA, however, showed significantly greater improvement with IAHA in pain on the 50-foot walk than placebo and more IAHA–treated patients (48%) had slight pain or were pain free than placebo-treated (33%) or naproxen-treated (37%) patients at 26 weeks after the treatment [34]. In studies using high MW HA in comparison with placebo, some degree of placebo effect was also observed; however, IAHA had better results than placebo for certain efficacy endpoints (WOMAC pain sub-score on walking [36] and efficacy durability by Kaplan–Meier analysis [29]). Even in several meta-analyses in which data from most of representative placebo-controlled trials of IAHA were combined, a definitive conclusion was not made and mixed results were presented [8, 9, 37, 38] like following: IAHA did not prove clinically significant efficacy and showed greater risk of adverse events than placebo [8]; small efficacy observed with IAHA might have been overestimated due to publication bias and high molecular weight HA was more effacious than low molecular weight HA but heterogeniety of the studies limited the definite conclusion [9]; IAHA had moderate efficacy compared with placebo until 10 weeks but not at 15 to 22 weeks [37]; or significant improvement in pain and functional outcomes were observed with IAHA but the efficacy was overestimated in low methodological quality of studies [38]. This inconclusive results may be mainly attributed to variety in the IAHA products, variable disease severity, difference in study designs, injection regimens, outcomes evaluation criteria, and rescue medication used in the studies included or not in the analyses. Further studies are needed to answer the question on the efficacy of IAHA considering various factors mentioned above.

Some limitations of the present study need to be addressed. First, two injections of placebo saline were mixed in the study arm. As HMWHA requires three weekly injections whereas XLHA only needs to be injected once, placebo injections in the study arm were inevitable to maintain double-blind status which was critical to maintain objectiveness of this study. Second, the period of 3 months follow-up may seem to be short. There have been some studies that evaluated the effect of hyaluronate in patients with knee OA about 3 months and the effect of HA is known to be shown between 5 and 12 weeks [39–41]. In addition, the extension of follow-up period could increase a drop-out rate, which might increase the bias obtaining optimal data in this kind of clinical trial. Based on these points, the evaluation period of this clinical trial was determined by a consensus with the government regulatory authority and IRBs of the participating institutions. Finally, 12 centers may seem to be too many. However, all investigators adhered to the protocol of this clinical trial without major violations. As this study evaluated a novel XLHA substance for a single injection therapy, multi-centers were needed to enroll the more than 300 subjects in a reasonable study period.

## Conclusions

The present study demonstrated that a single injection of XLHA was non-inferior to three weekly injections of HMWHA in terms of WBP reduction, and supports XLHA as an effective and safe treatment for patients with mild to moderate knee OA. Although this study has provided an evidence regarding the efficacy of IAHA, generalisation of the results of this study could be made after obtaining more supporting data from further researches that will focus on the durability of IAHA beyond 12 weeks and comparative study on the efficacy of variable IAHA preparations.

## Abbreviations

ADRs: Adverse drug reactions
AEs: Adverse events
BDDE: Butanediol diglycidyl ether
CI: Confidence interval
HA: Hyaluronan
HMWHA: High molecular weight HA
IAHA: Intra-articular HA
MW: Molecular weight
NSAIDs: Non-steroidal anti-inflammatory drugs
OA: Osteoarthritis
OMERACT-OARSI: Osteoarthritis Research Society International Standing Committee for Clinical Trials Response Criteria Initiative
WBP: Weight-bearing pain
WOMAC: Western Ontario and McMaster Universities Osteoarthritis Index
XLHA: Cross-linked HA.
